#  Preparation and Characterization of Three Tilmicosin-loaded Lipid Nanoparticles: Physicochemical Properties and *in-vitro* Antibacterial Activities

**Published:** 2016

**Authors:** Alwan Al-Qushawi, Ali Rassouli, Fatemeh Atyabi, Seyed Mostafa Peighambari, Mehdi Esfandyari-Manesh, Gholam Reza Shams, Azam Yazdani

**Affiliations:** a *Department of Pharmacology, Faculty of Veterinary Medicine, University of Tehran, Tehran, Iran. *; b *Department of Pharmaceutics, Faculty of Pharmacy, Tehran University of Medical Sciences, Tehran, Iran.*; c *Department of Avian Pathology, Faculty of Veterinary Medicine, University of Tehran, Tehran, Iran.*; d *Nanotechnology Research Center, Tehran University of Medical Sciences, Tehran, Iran.*

**Keywords:** Tilmicosin, Lipid nanoparticles, Stability, Mannitol, Antibacterial activity

## Abstract

Tilmicosin (TLM) is an important antibiotic in veterinary medicine with low bioavailability and safety. This study aimed to formulate and evaluate physicochemical properties, storage stability after lyophilization, and antibacterial activity of three TLM-loaded lipid nanoparticles (TLM-LNPs) including solid lipid nanoparticles (SLNs), nanostructured lipid carriers (NLCs), and lipid-core nanocapsules (LNCs). Physicochemical parameters such as particle size-mean diameter, polydispersity index, zeta potential, drug encapsulation efficiency (EE), loading capacity, and morphology of the formulations were evaluated and the effects of various cryoprotectants during lyophilization and storage for 8 weeks were also studied. The profiles of TLM release and the antibacterial activities of these TLM-LNPs suspensions (against *Escherichia coli* and *Staphylococcus aureus*) were tested in comparison with their corresponding powders. TLM-LNPs suspensions were in nano-scale range with mean diameters of 186.3 ± 1.5, 149.6 ± 3.0, and 85.0 ± 1.0nm, and also EE, 69.1, 86.3, and 94.3% for TLM- SLNs, TLM-NLCs, and TLM- LNCs respectively. TLM-LNCs gave the best results with significantly low particle size and high EE (p<0.05). Mannitol was the most effective cryoprotectant for lyophilization and storage of TLM-LNPs. The drug release profiles were biphasic and the release times were longer at pH 7.4 where TLM-NLCs and TLM-LNCs powders showed longer release times. In microbiological tests, *S. aureus *was about 4 times more sensitive than *E. coli* to TLM-LNPs with minimum inhibitory concentration ranges of 0.5-1.0 and 2-4 µg/mL respectively, and TLM-LNCs exhibited the best antibacterial activities. In conclusion, TLM-LNP formulations especially TLM-LNCs and TLM-NLCs are promising carriers for TLM with better drug encapsulation capacity, release behavior, and antibacterial activity.

## Introduction

Tilmicosin (TLM), a derivative of tylosin, is a macrolide agent for animal use particularly for treating respiratory diseases in poultry and cattle because of its accumulation in pulmonary tissues. It inhibits gram-positive bacteria including *Clostridium spp*., *Staphylococcus spp*., and *Streptococcus spp*., as well as some gram-negative bacteria including *Actinobacillus spp*., *Campylobacter spp*., and *Pasteurella spp*. Susceptibility of *Mycoplasma* can be quite variable (1). In general, gram-negative* Enterobacteriaceae *are resistant to TLM at low doses, and it needs high doses to penetrate them; therefore, it is not used for these organisms (2, 3). Currently, soluble TLM phosphate is used, but this form exhibits problems of low potency, poor safety, and relatively low systemic bioavailability; so, development of a novel delivery system for TLM is warranted (4). 

Recent advances in nanoparticulate drug delivery systems display a great potential for the proper dosing and targeting the active molecules. These carriers protect active molecules from chemical and biological degradation. Among several nanoparticle systems, lipid carriers have gained a lot of interest and developed for various administration routes (5). By these carriers, not only the drug is delivered to the target site properly, but also the dosage regime can be modified and reduced its adverse effects (6), the lipid carrier systems offer a number of advantages including low toxicity, good biocompatibility, great kinetic stability, and appropriate production scalability (oral, dermal, intravenous) (7, 8). Several types of lipid nanoparticles (LNPs) have been designed in the last 20 years; the most important ones are solid lipid nanoparticles (SLNs), nanostructured lipid carriers (NLCs), lipid drug conjugates (LDCs), and lipid-core nanocapsules (LNCs) (8)**. **SLNs have gained a great attention as a drug delivery system when it was first developed in 1991 by Muller (9). This system consists of the entrapped drug in biocompatible lipid core and surfactant at the outer surface (10). To reduce the shortcomings associated with SLNs, a new model of LNPs, namely NLCs, was developed consisting of a lipid matrix with a particular nanostructure (11)**. **This nanostructure increased drug encapsulation efficiency and drug stability during storage. While SLNs consist of solid lipids, NLCs include some liquid lipids, which make imperfections in the lipid matrix (12). Recently, nanocapsules such as LNCs were developed, that is a vesicular system in which a drug is restricted to a cavity bounded by a polymer film (13)**. **LNCs are considered as a hybrid delivery system with a core containing medium-chain triglycerides enveloped by a polymeric membrane (14). This structure provides some more advantages such as higher drug encapsulation capacity, higher protection from drug degradation, and lower burst release (15).

To improve pharmacological properties of TLM, this study as a preliminary project was aimed to formulate three novel TLM- loaded lipid nanoparticles (SLN, NLC and LNC) and to characterize their physicochemical properties, *in-vitro* drug release behavior, and antibacterial activities.

## Experimental

*Preparation of TLM-SLNs*

TLM-SLN suspension was prepared by hot homogenization followed by ultrasonic technique. 3.6 g of Hydrogenated castor oil heated in water bath and, then, 0.40 g of TLM base (97.3% provide by Razak Pharmaceutical Co., Tehran, Iran) were dissolved in the melted lipid. 40 mL of 5% Poly Vinyl Alcohol (PVA) 87%-90% hydrolyzed) solu­tion was preheated, poured into the lipid phase, and maintained under a magnetic stirrer at 250 rpm for 5 min. The obtained emulsion was homogenized in an Ultraturrax (IKAT18, Germany) at 16,000 rpm for 5 min and then sonicated (Misonix, USA) for 30min by using the 13 mm microprobe with amplitude 35%, then it was poured into cold water to yield a TLM-SLN suspension. This suspension was washed and collected with filtration using Millipore centrifuge tubes and was centrifuged at 4000 rpm for 45 min at 4 °C and then, was washed three times with distilled water to remove excess PVA and un-entrapped TLM (4, 16).

*Preparation of TLM-NLCs*

TLM-NLC suspension was prepared by hot homogenization technique (16, 17). A 2.85 g of Compritol 888 ATO (Glyceryl behenate-Gattefossé, France) and 0.75 g of Sesame oil (Sigma Aldrich, Germany) were melted at 80 °C, then 0.40 g of TLM was added to the lipid phase dispersed in aqueous phase containing a mixture of surfactants, 5% Poloxamer 407 (Synopharm, Germany), and 5% Tween 80 (Merck, Germany) simultaneously prepared at 80 °C and then, the mixture wasstirred at 750 rpm for 10 min. The obtained pre-emulsion was homogenized at a temperature 5 °C to 10 °C higher than the melting point of the bulk lipid using a high-shear homogenizer at 2000 rpm for 1 h in an Ultraturrax (IKAT18, Germany). TLM-NLC suspension was removed from water bath and mixed until cooled**.**

*Preparation of TLM-LNCs*

TLM-LNC suspension was prepared according to method described by Jager (18) based on the principle of interfacial deposition reported by Fessi (19). The organic phase containing 0.125 g of Eudragit S 100 (Röhm Pharma, Germany), 0.070 g of Span 80 (Sigma Aldrich, Germany), 0.050 g of TLM, and 0.325 g from Coconut oil (Sigma Aldrich, Germany) were dissolved in 30mL of Acetone and kept for 60 min under stirring at 40 °C. This mixture was then poured into 53 mL of an aqueous solution containing 0.070 g of Tween 80 and maintained under moderate stirring at 40 °C. After 10 min, the mixture was evaporated using rotary evaporator to eliminate the Acetone.

Blank suspensions of SLNs, NLCs, and LNCs were considered as control. All suspensions were made in triplicate**.**

*Physicochemical characterization*

Size-mean diameter (MD), zeta potential (ZP), and polydispersity index (PDI) of TLM-SLNs, as well as TLM-NLCs and TLM-LNCs were determined by photon electron spectroscopy using Zetasizer (Malvern Instruments, UK). Before measurement, the samples were diluted appropriately with distilled water (for LNC was diluted with an aqueous solution of 10 mM NaCl). 

*Morphology analysis*

The morphological characteristics of freeze-dried TLM-LNPs were determined by scanning electron Microscopy (SEM). Briefly, 5 mg of each sample was suspended in 1 mL of distilled water, and 2 μL of the suspension was placed on a cover slide and dried at room temperature. The slide was coated with gold under vacuum using a sputter coater for 10 min, and then was analyzed. 

*Freeze drying (lyophilization)*

To lyophilize TLM-LNP suspensions, they were diluted with different cryoprotectant solutions including mannitol, sucrose, lactose, and sorbitol (5%, w/v). Then, they were pre-frozen for 12 h at −20 °C and subsequently lyophilized at −80 °C for 24 h in a freeze-dryer. Finally, the powders were reconstituted with distilled water, and then, their physicochemical characterizations, encapsulation efficiency, and loading capacity were measured freshly.

*Stability study*

TLM-LNP powders were stored in glass vials at room (25 ± 1 °C) and refrigeration (4 ± 2 °C) temperature for a period of 60 days. All samples were analyzed at appropriate time intervals (0 time, 4^th^ weeks and 8^th^ weeks) by measurement of their physicochemical characterizations, encapsulation efficiency, and loading capacity.

*Drug encapsulation*

Drug encapsulation efficiency (EE) and loading capacity (LC) were determined using a UV-visible spectrophotometer (Aquarius, Cambridge, UK) by indirect method (20, 21) for suspensions compared to the freeze-dried ones to detect any leakage of drug from nanoparticles during lyophilization.To determine the TLM content in TLM-LNPs freeze-dried, 10 mg of each formulation was dissolved in chloroform and analyzed directly at 291 nm. For TLM-LNP suspensions, volumes of 1 mL (containing an equivalent amount of TLM to 10 mg of freeze-dried) were centrifuged at 12,000 rpm for 20 min at 4 °C, and the amount of free TLM in the aqueous medium was determined. Then, drug encapsulation rate was spectrophotometerically determined. EE and LC were calculated using the following equations:

% EE=Weight of TLM added - Weight of free TLM Weight of TLM added *100

$\%\mathrm{LC}=\frac{\mathrm{Weight of TLM used}-\mathrm{Weight of free TLM}}{\mathrm{Weight of SLNs},\mathrm{NLCs or LNCs}}\mathrm{*100}$

*In-vitro release study*

*In-vitro* release studies were performed by dialysis bag method using membranes with MW cut-off, 12,000-14000 KDa. Exactly weighed amounts of LNP powders with lyophilized mannitol, LNP suspensions and TLM standard suspensions equivalent to 3 mg TLM were placed in dialysis bags (containing 5 mL of donor solutions of phosphate buffer saline (PBS) at pH 7.4 or 0.1 M HCL at pH 1.2), sealed and dialyzed against 45 mL of PBS or 0.1 M HCL in a 50 mL beakers which were placed in a thermostatic shaker at 37 °C ± 0.5 °C at a rate of 100 rpm. Aliquots were withdrawn from the receiver solutions at pre-determined intervals up to 250 h and the samples were replaced by fresh buffer. TLM amounts in the release mediums were determined using a UV-visible spectrophotometer at 291 nm (21). 

*Antimicrobial activity study*

To determine the antibacterial activity of TLM-LNP (suspensions and powders), the well diffusion test was carried out by using bacterial strains;* E. coli* as a gram-negative pathogenic strain and *S. aureus* as a gram-positive pathogenic strain were poultry isolates, provided by the Lab. of Dept. Poultry Diseases, Faculty of Veterinary Medicine, University of Tehran, Iran. The bacterial suspensions were transferred onto the surface of Muller-Hinton agar plates (Quelab; Quebec, Canada). Wells with 8mm diameters were prepared in the solid agar medium. Aliquots of 100 µL of each aqueous concentration were added into the wells for different TLM-LNP formulations (powders and suspensions). After incubation for about 24 h at 37 °C, the zones of inhibition (mm) were measured. 

*Determination of MIC and MBC*

To determine MIC and MBC of TLM-LNP (suspensions and powders) and standard TLM against *E. coli* and *S. aureus*, broth dilution method was used. A TLM stock solutions of 32 µg/mL for different TLM-LNP formulations (powders and suspensions) and standard TLM were prepared, and then further diluted to yield concentration range of 0.5 to 32 µg/mL (depending on their drug loading) in 4 mL of Muller–Hinton broth (Quelab; Quebec, Canada). To test MIC, after 24 h incubation at 37 °C, the test tubes were examined for possible bacterial turbidity; the MBC was measured by sub-culturing from MIC broth tubes onto fresh agar plates. 

*Statistical analysis*

All data were expressed as mean ± SD. The data were analyzed by SPSS 16.0 using one-way analysis of variance (ANOVA) followed by post hoc multiple comparisons and P<0.05 was set as significant level.

## Results and discussion

*Preparation of TLM-LNPs and Physicochemical characterizations*

Hot homogenization presented many advantages such as rapid and easy to perform (22), no use of organic solvents (23), and yielding smaller MD and PDI values. Ultrasonication was also contributed to produce smaller MD along with homogenization (24). TLM-LNC which was prepared on principle of interfacial deposition, provided much better physicochemical properties (25).

The mean values of MD, ZP and PDI, as well as LC and EE of the prepared formulations are shown in Table 1. MD values of freshly prepared suspensions demonstrated significant differences (p<0.05), but in general, they were in nano-range scale (between 85-186 nm). Among all suspensions, TLM-LNC showed the lowest MD values; 85.0 ± 1.00 nm, while TLM-NLC and TLM-SLN showed higher values; 149.6 ± 3.0 nm and 186.3 ± 1.50 nm, respectively.

PDI values of all formulations were found to be below 1.0 suggesting that they had monodispersed system, which remained stable (value closer to zero refers to increased homogeneity in the MD). PDI values were below 0.3 for all formulations. PDI values were low, but TLM-LNC values (0.12 ± 0.01) were found to be significantly (p<0.05) less than other suspensions, while for TLM-SLN and TLM-NLC, they were 0.13 ± 0.05, 0.28 ± 0.05 respectively which indicate more homogeneity of TLM-LNC formulation**.**

ZP values of all prepared formulations were found to be negative; TLM-NLC was significantly (p<0.05) more negative (-29.3 ± 2.51 mv). It predicts high particle stability and limits aggregation with prolong storage, while TLM-LNC and TLM-SLN had less negative charges (-17.3 ± 2.08 and -18.9 ± 1.26 mv, respectively).

It is believed that the MD is one of the most important parameters for evaluating the stability of colloidal systems (26). The small MD of TLM-NLCs in comparison with TLM-SLNs could be due to the less crystalline structure of NLC (27) and combining the surfactants would reduce the MD because of synergism (28). Using high concentrations of Tween 80 in preparation of TLM-LNC and TLM-NLC promote the production of smaller nanoparticles (29, 30)**. **Tween 80 is essential to stop coalescence of the colloids because of high interfacial pressure between the organic and aqueous phase, which consequently leads to decrease in PDI values**. **The existence of Tween 80 was enough to coat the shell region and prevent the coalescence due to a steric stabilizing effect (25). Moreover, increasing Poloxamer 407 concentrations in TLM-NLC resulted in lower MD values, which led to preventing the coalescence of the droplets (31).

**Table 1:** Physicochemical properties of freshly prepared TLM-LNP suspensions (SUS), formulations after lyophilization with mannitol (MAN), and without adding cryoprotectant (WAC). MD: particle size-mean diameter, PDI: polydispersity index, ZP: zeta potential, EE: encapsulation efficiency, LC: loading capacity

**Parameter**	**Formulation**	**TLM-SLN**	**TLM-NLC**	**TLM-LNC**
LC (%)	SUS	6.43±0.09 C	8.50±0.04 B	9.50±0.05 A
	MAN	6.35±0.05 C	8.33±0.06 B	9.30±0.04 A
	WAC	6.40±0.04 C	8.10±0.05 B	9.00±0.05 A
EE (%)	SUS	69.1±2.80 C	86.3±2.30 B	94.3±2.04 A
	MAN	66.3±2.67 C	86.5±2.17 B	94.0±3.60 A
	WAC	65.2±3.00 C	81.3±0.98 B	90.0±2.43 A
ZP (mV)	SUS	-18.9±1.26 Ba	-29.3±2.51 Aa	-17.3±2.08 Ca
	MAN	-15.6±3.21 Ba	-23.5±1.13 Ab	-16.3±2.51 Ba
	WAC	-9.03±0.20 Ac	-10.8±2.61 Ac	-10.3±2.08 Ab
PDI	SUS	0.13±0.05 Bb	0.28±0.05 Ab	0.12±0.01 Cc
	MAN	0.13±0.001Cb	0.27±0.03 Ac	0.22±0.01 Bb
	WAC	0.27±0.002 Ca	0.50±0.05 Aa	0.26±0.005 Ba
MD (nm)	SUS	186.3±1.50 Ac	149.6± 3.0 Bb	85.0±1.00 Cc
	MAN	193.0±2.64 Ab	156.6±7.63 Bb	116.6±7.63 Cb
	WAC	252.6±11.0 Ba	288.3±27.5 Aa	153.3±12.5 Ca

Data are expressed as mean±SD, n=3.

Different capital letters in horizontal and small letters in vertical values indicate significant differences (p<0.05).

**Table 2:** Stability of physicochemical properties of TLM-LNP powders lyophilized with mannitol (MAN) or without adding cryoprotectant (WAC) stored at 4 °C and 25 °C for 8 weeks

**Parameter**		**TLM-SLN**		**TLM-NLC**		**TLM-LNC**	
		**4 °C**	**25 °C**	**4 °C**	**25 °C**	**4 °C**	**25 °C**
LC (%)	MAN	6.50±0.02	6.50±0.04	8.50±0.01	8.50±0.03	9.50±0.02	9.50±0.02
	WAC	6.40±0.04	6.40±0.01	8.10±0.04	8.00±0.02	9.00±0.03	9.00±0.02
EE (%)	MAN	66.3±5.67	66.30±5.67	85.0±5.00	85.0±5.00	95.0±3.00	94.0±1.60
	WAC	65.1±2.46	64.16±1.60	81.0±1.00	80.3±0.57	90.0±5.00	90.0±5.00
ZP (mV)	MAN	-17.0±1.32	-15.16±1.15	-23.00±2.64	-23.00±2.64	-17.00±2.64	-16.66±2.88
	WAC	-8.9±0.23	-4.33±4.04	-10.00±2.00	-7.667±3.78	-10.66±1.15	-10.66±1.15
PDI	MAN	0.138±0.001	0.137±0.002	0.276±0.034	0.275±0.034	0.231±0.01	0.231±0.01
	WAC	0.270±0.005	0.300±0.008	0.516±0.055	0.513±0.057	0.270±0.004	0.280±0.009
MD (nm)	MAN	192.3±2.51	192.3±2.51	156.6±7.63	158.3±7.63	118.6±5.85	117.3±6.80
	WAC	254.3±5.13	273.3±5.77	296.6±22.5	306.6±20.2	156.3±6.35	163.6±10.6

Data are expressed as mean±SD, n=3.

**Table 3:** The MIC and MBC values (µg/ml) of tested formulations against *S. aureus* and *E. coli* bacteria

**Bacteria**		**TLM-SLN** **powder**	**TLM-SLN** **suspension**	**TLM-NLC** **powder**	**TLM-NLC** **suspension**	**TLM-LNC** **powder**	**TLM-LNC** **suspension**	**TLM**
*S. aureus*	MIC	1	1	0.5	0.5	0.5	0.5	1
	MBC	4	4	3	2	3	2	4
*E. coli*	MIC	4	4	2	2	2	2	4
	MBC	12	12	10	9	10	9	12

**Figure 1:**
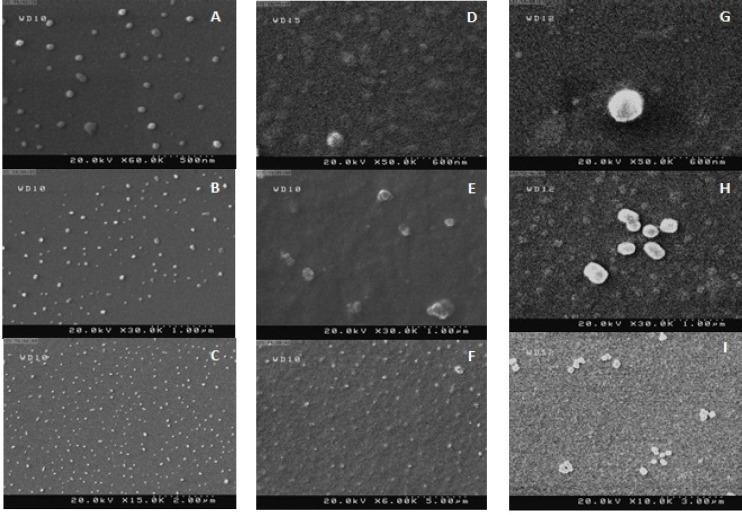
SEM graphs of TLM-LNPs lyophilized with mannitol. **A**, **B **and **C: **TLM-SLNs; **D**, **E **and **F**: TLM-NLCs; **G**, **H **and **I**: TLM-LNCs

**Figure 2:**
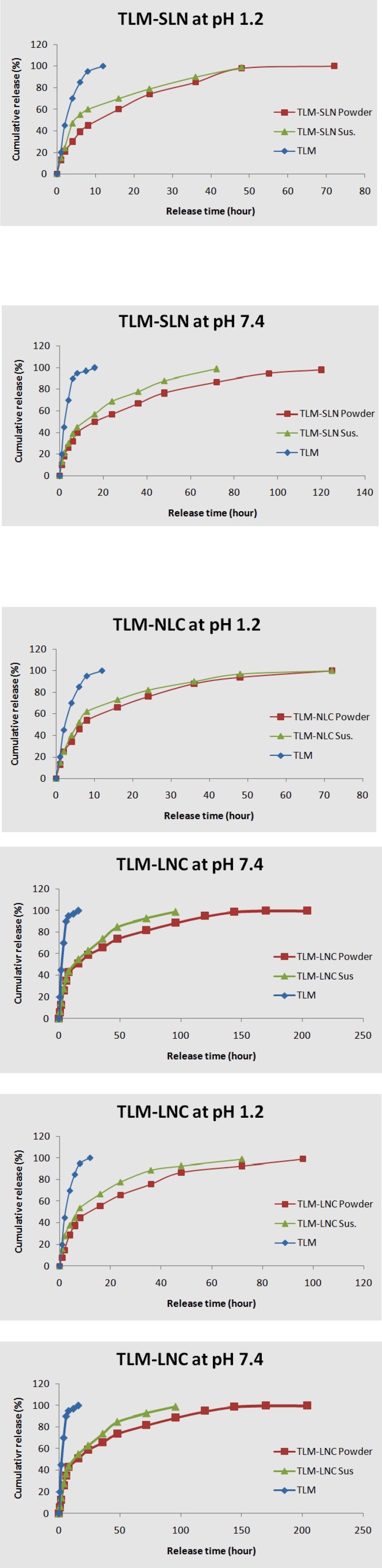
*In-vitro *release profiles of different TLM-LNPs.

**A. **TLM-SLN in 0.1 M HCl at pH 1.2, **B**. TLM-SLN in PBS at pH 7.4

**C. **TLM-NLC in 0.1 M HCl at pH 1.2, **D. **TLM-NLC in PBS at pH 7.4

**E. **TLM-LNC in 0.1 M HCl at pH 1.2, **F. **TLM-LNC in PBS at pH 7.4

**PBS: **Phosphate Buffer Solution, **HCL**: Hydrochloric acid, **Sus: **Suspension.

The relatively high PDI values of NLC are in accordance with Lasoń *et al.* (2013), as they found that these values were in the highest for Compritol 888 ATO lipid matrix (26)**. **The narrow PDI (less than 0.5) of TLM-SLN and TLM-NLC have been reported to be achieved at high homogenization pressure (24, 32). Low values for PDI were found with TLM-LNCs because of the high efficiency of interfacial deposition method in preparation of homogenous formulations (15).

The increasing of surface charges (±) refers to the level of repulsion (stability) among near-similarly charged particles (28). ZP is deeply dependent on the nature of coating (23). The increased negative charge of TLM-NLCs and TLM-LNCs could be due to oleic acid traces found in Tween 80, which make a dense surfactant film (33) and the high surfactant concentrations (5%) used (34). With regard to TLM-LNCs, the high ZP value may also be related to carboxylic group of methacrylic acid residues in the enteric Eudragit S100 backbone (35). The higher PVA concentrations in TLM-SLNs possibly lead to high density of PVA at the oil-water interface among suspension droplets and may increase the thickness of the particles shell. This coat shell is potentially able to shield the surface charge of the preparation and consequently, it is reasonable to expect less ZP values (4). Hence, the applying liquid lipids and polymers of Eudragit in TLM-NLCs and TLM-LNCs, and more than one surfactant are critical factors to get better physical qualities. 

*Morphology*

SEM of TLM-SLN, TLM-NLC, and TLM-LNC are shown in Figure 1. The size of particles in dry powders of lyophilized formulations with mannitol was found within the nano-scale. TLM-LNC particles were relatively spherical and regular in shape, while for TLM-NLC particles were spherical in shape with platelet-like shape. The TLM-SLN particles were confirmed to be with smooth texture of surface with regular 

spherical form. A little aggregation may result because of gluing of nanoparticles together due to the lipid structure of the carriers and the drying process of sample during its preparation at ambient temperature previous to SEM examination (36, 12). 

*Freeze drying (lyophilization)*

The physicochemical properties as well as LC and EE values of TLM-LNPs formulations before and after lyophilization are shown in Table 1. Little changes in physiochemical properties of lyophilized formulations in comparison to suspensions were achieved after lyophilization with mannitol. They yielded fine powders and homogenous solutions after reconstruction with a significant increase (p<0.05) in MD (193.0 ± 2.64, 156.6 ± 7.63 and 116.6 ± 7.63 nm for TLM-SLN, TLM-NLC and TLM-LNC, respectively); and with small changes in PDI and ZP values. LC and EE values were almost the same as their values before lyophilization; but drastic changes in values of all physicochemical parameters were observed with other cryoprotectants (sucrose, lactose, and sorbitol) and those lyophilized without adding cryoprotectant. 

Due to generation of stresses during lyophilization of preparations, cryoprotectants are added to protect the particles (37). Mannitol is usually used as lyophilized agent in pharmaceutical industry due to its high stability (38). It is used at concentration of 5% to achieve less variation in MD values (39). However, a small increase in MD values after lyophilization is expected (40)**. **The conservation of MD and PDI values after lyophilization is a good sign of a successful lyophilization process. Calculation of ZP is a good technique to evaluate any possible interface between the cryoprotectant and particles after lyophilization. Through the freeze-drying, crystallized water content of electrolytes will decrease the ZP, which may lead to nanoparticles aggregation (36). Decreased ZP values may also occur as result of particles surface masking due to hydrogen bonding between OH groups of cryoprotectant agent and the particle surfaces (40). LNCs contain a thin polymeric envelope, which may not resist the stresses of lyophilization adequately; so the agglomeration of LNCs and loss of drug are expected. A fully redispersion of LNC with keeping of their MD and PDI values can be achieved when cryoprotectant were used at a concentration of 5% (40). Lower physical quality after lyophilization are expected but with using effective cryprotectant agent such as mannitol and polymeric envelop these changes can be limited. 

*Storage stability*

The physicochemical characterizations as well as LC and EE values of TLM-LNPs powders (lyophilized with mannitol or without) after storage for 8 weeks are shown in Table 2. All lyophilized formulations showed good redispersibility in water at the end of the 8th week. The MD values were in the range of 117-306 nm and PDI values between 0.136-0.513 after storage for 8 weeks at 25 ± 1 °C. The results indicated that formulations lyophilized with mannitol had better physicochemical characterizations as well as LC and EE. Therefore, these formulations were chosen for next studies. Mannitol led to production of a fine powder and a solution with better properties at different period of storage. The highest value which indicated less stability, were achieved with formulations that dried without adding cryoprotectant. 

The shelf life of colloidal preparations can be enhanced by lyophilization since it increases the chemical and physical stability over extended periods**. **Temperature seems to be very important for prolonged stability. 4 ± 2 °C is most favorable storage temperature, while 25 ± 1 °C is suitable for long-term storage (24)**. **

The continuous reduction in physical quality in some prepared formulations is due to storage at high temperature. Increasing the temperature enhances the kinetic energy, with a reduced ZP values which leads to fusion of LNPs, while storage at low temperature, ZP values remains stable (41, 34). Our results regarding TLM-LNPs, lyophilized without adding cryoprotectants, agree with Dixit *et al*. (2011). They studied physical stability of indomethacin-LNCs, after storage for 12 months at 25 °C and 4 °C. When they stored the powders at 25 °C, they lost 8.5% of indomethacin after 2 months, but the loss was just 9.3% at the end of 12th month after storage at 4° C (36)**. **However, lyophilization made increases in MD values, which reached to two folds more than initial values as result of particles clustering; but all physicochemical properties seem to be highly effected with increase storage temperature degree. 

*Drug encapsulation*

The maximum EE and LC suspensions values (checked directly after preparation) was reported with TLM-LNC (p<0.05) and the minimum values was found for TLM-SLN. After lyophilization, mannitol relatively yielded better values. The most reduction in EE and LC values was achieved with formulation lyophilized without adding cryoprotectants. The values are shown in Table 1. 

It is very important to calculate encapsulated drug because it determines the release pattern (42) and therapeutic efficacy (8)**. **Low drug loading of TLM-SLN may be due to the use of high concentration of PVA, which is used to decrease the MD values; this leads to high diffusion of TLM into aqueous phase during preparation and lyophilization (4)**. **NLC formulation, which is prepared by both solid and oily lipid, does not recrystallize and keeps its amorphous nature, which provides many imperfections space to encapsulate more TLM (28). NLC has been formulated to make particles in which the oil is in the core of a solid lipid, which in turn, it makes an increase in LC because the drug is dissolved in the oil and bounded by solid lipid (43). High drug loading of LNCs could be due to the fact that Eudragit polymer provides enough coating, and less pore and leakage of the TLM happens because of rapid formation of a boundary around the droplets (35). In addition, the lipophilic nature of the core in LNCs leads to increase in its drug encapsulation (44). Mannitol may play a role in encapsulation efficiency for all prepared formulations, and it is applied in many encapsulation efficiency studies (45). 

The type of lipid carrier determines its loading capacity, which can increase by using hybrid delivery system composed of lipid and polymer such as TLM-LNC formulation in the present study. 

*In-vitro release*

 The *in-vitro *release profiles of TLM for different LNPs were depicted in Figure 2. The standard TLM achieved 97% and 100% drug release within 12 h at pH 7.4 and 1.2, respectively; but other formulations presented biphasic release model with an initial burst release followed by sustained release. TLM-LNP suspensions showed faster initial burst release in comparison to their powders. TLM-NLC powder showed an initial burst release of 15% at pH 7.4 within first 2 h followed by a constant sustained release for 200 h, while TLM-LNC powder at pH 7.4 achieved the least initial burst release among all tested formulations, which was 13% release within first 2 h followed by constant sustained release for 200 h. 

Drug release rate from LNPs was determined by drug partition between the lipid phase and the aqueous environment in the dialysis bag, then by diffusion of the drug through the membrane. The releasing profiles depend on formulation method since it has been noted that the higher temperature and surfactant concentration improved the drug solubility (10). Battaglia and Gallarate (2012), and also Attama *et al. *(2012) reported that the drug release pattern depends on its affinity to lipid matrix because it could affect the viscosity of LNP matrix, which may play an important role in controlling the release pattern (8, 12, 46). The standard TLM release behavior is only dependent on diffusion through the dialysis bag (47). Since TLM is a Lewis base, it is highly hydrophobic at basic conditions; while in acidic environment, TLM can produce salts which enhance its aqueous solubility and increase its release (4). Low pH medium can accelerate lipid matrix hydrolysis and produce high concentration of TLM in the medium (4, 34, 28). 

The most possible reason for faster burst release of TLM-LNP suspensions may be due to found of poor incorporated TLM on particles surface. However, the release behavior can be modified as a function of incorporated lipid, surface modification, and surfactant (5, 48, 49). In suspensions, where the TLM is homogeneously dispersed, the release profile depends on diffusion and erosion of the lipid matrix in sink circumstances. Our findings are agreeing with Xie *et al*. (2011) and with Chen et al. (2014), when they reported the faster burst release of drugs from the suspensions at first hours compared with SLN powders (50, 4). In addition to poor incorporated drug in NLC, another reason that could explain its release behavior is the oily lipid found in outer shell which locates some of the drug on shell and contributes the initial burst (12), while the following slow release pattern of NLC may be due to its higher loading capacity (8). NLC release profile almost takes the drug-core model according to Fick’s law of diffusion because the lipid surrounds the drug as a membrane (51). The presence of coating polymer in TLM-LNC makes the release profile controlled by diffusion of the TLM from the core to the polymeric coat, where it acts as a releasing barrier. Therefore, the TLM solubility and its diffusion in polymer membrane will be a limiting factor in its release (5). It seems that Eudragit S 100 polymer could be an appropriate option to formulate sustained release TLM-LNPs (27, 44). Moreover**, **the sustained release nature of LNCs could be as result of the variation of its core viscosity and the particle surface. It also noted that the increase in the Span 80 concentration improved the resistance to the pro-drug diffusion by increasing the core viscosity (22). Select suitable formulation method, preparation matrix, surfactant, and particles surface modification were critical factors in improvement of behavior release for TLM-NLC and TLM-LNC formulations. 

*Antimicrobial activity*

 The antibacterial activity (as zone of inhibition-mm) of different formulations showed that *S. aureus *was more sensitive to prepared formulations. Among all the tested formulations, the TLM-LNC powder and TLM-LNC suspension exhibited higher significant (p<0.05) antibacterial activities against the tested bacteria in terms of zone of inhibition at 0.5 μg/mL followed by TLM-NLC powder and TLM-NLC suspension, while TLM-SLN powder, TLM-SLN suspension, and standard TLM achieved the less activity (1 μg/mL). However, the first result which indicated on *E. coli *sensitivity was achieved at concentration of 4 μg/mL of TLM-LNC powder and suspension, they were significantly (p<0.05) more active in comparison to other formulations. In general, all the formulations showed antibacterial activity at concentration 8 μg/mL, thereafter the bacterial growth rebounded, suggesting that antibacterial activity decided dependently on drug encapsulation and particles size. 

MIC and MBC values (Table 3.) of TLM-SLN and standard TLM against *E. coli *was about three-four fold more than *S. aureus *values. This means that loading of TLM in SLN does not lead to enhance antimicrobial activity of TLM, while TLM-NLC and TLM-LNC succeeded in enhancement of antibacterial activity of TLM, but their values showed some variations between suspensions and powders. Using of nanoparticles to entrap antibacterial agents may improve their activity due to their sustained release and higher ability to penetrate the bacterial cell wall due to their small size and their hydrophobic structure, which is like to gram-negative cell wall bacterial (52, 53). The blank control formulations had no antibacterial activity, so the enhanced antibacterial efficacy was due to the ability of these formulations to deliver the TLM molecules efficiently to the site of action**. **The higher antibacterial activities of TLM-LNC powder and suspension indicated that the entrapment of TLM in these carriers was more efficient than other carriers. According to the literature, using LNC with a mixture of Eudragit and PLGA for loading ciprofloxacin achieved stronger inhibition for *P. aeruginosa *and *S. aureus*, which was due to their smaller size and higher loading capacity; so it could be internalized by bacterial cells more efficiently (15)**. **The increased antibacterial activity TLM-NLC may also be due to a higher extent of the oily lipid and TLM concentrations, since it has been reported that prepared NLC with Tween 80 induces increased antibacterial activity against *E. coli*. (54), while moderate antimicrobial activity of TLM-SLN in comparison to other formulations especially with regard to TLM-SLN powder may be explained by their lower loading capacity and relatively high particles size (53). Our results are agreeing with Chen *et al*. (2014) and Wang *et al. *(2012) with regard to TLM-SLN where they reported the same MIC and MBC values as that of the standard TLM, the smaller particles size led to lower their MIC and MBC values of TLM-SLN suspension in comparison with TLM-SLN powder (4, 20). The less antibacterial activities against *E. coli*, especially for TLM-SLN and standard TLM at low doses could be attributed to the presence of the outer membrane in which large sized particles difficultly cross through it; so they need high doses to penetrate *E. coli *(2, 53). In general, activity of the prepared formulations was related to the type, loading, particles size values, and tested bacterial species. It was demonstrated that entrapped TLM in the formulations was in an active form, and thus formulation processes did not substantially deteriorate the drug activity. However, LNC carrier seems to be a promising formulation for TLM against both bacteria. 

## Conclusions

 TLM effectively loaded in lipid nanoparticles and the best physicochemical properties, drug release behavior as well as antimicrobial activity were achieved by TLM-LNCs followed by TLM-NLCs. Mannitol as a cryoprotectant provided the best results in stability tests. TLM-LNCs suggest more efficient treatment in comparison to the conventional TLM, even though more studies are needed. 
